# Stigma Experiences, Mental Health, Perceived Parenting Competence, and Parent–Child Relationships Among Lesbian, Gay, and Heterosexual Adoptive Parents in the United States

**DOI:** 10.3389/fpsyg.2020.00445

**Published:** 2020-03-30

**Authors:** Rachel H. Farr, Cassandra P. Vázquez

**Affiliations:** Department of Psychology, University of Kentucky, Lexington, KY, United States

**Keywords:** adoption stigma, homonegative microaggressions, lesbian and gay, mental health symptoms, parent–child relationships, parenting competence

## Abstract

Adoptive parents often face stigma related to “non-traditional” family structures. Lesbian and gay (LG) adoptive parents often face additional stigmatization based on sexual identity, which in turn may negatively affect parents’ mental health. Despite controversy about LG parenting, research demonstrates that family processes are more strongly associated with individual outcomes than family structure. Thus, family systems and minority stress theories provided our conceptual foundation in examining how adoptive LG parents’ stigma experiences were associated with mental health, parenting competence, and parent–child relationships. Participating families (*N* = 106; *n* = 56 LG parent families) were originally recruited from five US domestic private infant adoption agencies and completed two waves of data collection (W1, W2; 91% retention) when children were preschool-age (*M*_age_ = 3.01 years) and school-age (*M*_age_ = 8.36 years), respectively. Data for the current study are largely drawn from W2. Via Qualtrics, parents completed assessments of mental health symptoms, adoption stigma, and perceived childcare competence. LG parents also reported on their experiences of homonegative microaggressions, and children responded to a measure about their relationships with parents. No significant differences emerged as a function of parental sexual orientation and gender except that lesbian mothers, heterosexual mothers, and gay fathers all reported higher parenting competence than heterosexual fathers. Although parents’ mental health did not significantly predict parent–child relationship quality, parents’ perceived competence and LG parents’ current homonegative microaggression experiences did (e.g., greater competence, greater closeness; more microaggressions, lower closeness). Consistent with our conceptual framework, our results—derived from parent and child reports—demonstrate that although adoptive and LG parent families experience stigma, family processes (rather than structure) are most associated with individual outcomes. Researchers, policy makers, and practitioners should work together to employ identity-affirming practices to reduce stigma and support adoptive family functioning and well-being.

## Introduction

Despite controversy, lesbian and gay (LG) adoptive parents in the United States (US) have increased in number and visibility; in fact, same-gender couples appear up to seven times more likely to have adopted children than different-gender couples (Goldberg and Conron, 2018). Regardless of ongoing debate about LG parenting, research supports that family processes (e.g., relationship dynamics between family members) are more strongly associated with individual outcomes than family structure (e.g., the number of parents, relationship status, etc.), including LG adoptive parent families (Lamb, 2012; Farr, 2017). Even so, in the US, adoptive parents often face stigma (e.g., concerns about parenting ability; Miall, 1987) related to “non-traditional” family structures (i.e., differing from married heterosexual parents with biologically related children), and LG adoptive parents often face additional stigma based on sexual identity (Goldberg, 2009; Herek, 2010; Goldberg and Smith, 2014; Lo et al., 2019). For example, the question of whether same-gender couples could raise children as effectively as do different-gender couples was a central debate in the ruling for marriage equality in the US, a ruling that held important legal implications about whether (married) same-gender couples could jointly adopt children (American Psychological Association [APA], 2015; Obergefell v. Hodges, 135 S. Ct. 2584, 2015).

Stigma felt by parents about their family composition may in turn negatively affect their mental health as well as perceived competence in parenting through internalized homophobia (Herek and Garnets, 2007; Herek, 2009; Newcomb and Mustanski, 2010; Robinson and Brewster, 2014). Research has also demonstrated that internalized stigma and stress may affect relationship quality among same-gender couples (Otis et al., 2006; Frost and Meyer, 2009), so it is possible that stigma could also be relevant to other family relationships, such as between parents and children in LG adoptive parent families. As such, family systems theory (Cox and Paley, 1997) and minority stress theory (Meyer, 2003) provided our conceptual foundation to examine how adoptive and LG parents’ stigma experiences were associated with mental health, parenting competence, and parent–child relationships. From family and minority stress perspectives, it is not surprising that contextual effects from both adoption stigma and homophobia can negatively affect parents’ mental health (Battle and Ashley, 2008; Frost and Meyer, 2009; Boss et al., 2016; Calzo et al., 2019; Goldberg et al., 2019). Our purpose here was to examine how stigma related to adoption and sexual orientation experienced by adoptive LG parents in the US may be associated with parent adjustment and their young children’s reports of parent–child relationship quality. Utilizing both parent and child reports is a major strength of this study and a unique contribution to the literature. This unique sample of families diverse in parental sexual orientation (i.e., lesbian, gay, and heterosexual parents), yet all with young adopted children, provided us an opportunity to investigate who might be more at risk or protected from experiences of stigma.

There is overwhelming consensus in the scholarly literature that children in LG parent families (including those formed through adoption) are well-adjusted and show high-quality parent–child relationships (Erich et al., 2009a, b; Patterson, 2017; Calzo et al., 2019; McConnachie et al., 2019). Indeed, few differences in outcomes have been uncovered in comparing children in LG parent families versus those in heterosexual parent families (Bos et al., 2016; Farr, 2017; Patterson, 2017; Calzo et al., 2019). LG parents, including LG adoptive parents, demonstrate high levels of effectiveness and competence in their roles as parents and healthy psychological adjustment as compared with their cisgender heterosexual parent counterparts (Bos et al., 2004a; Goldberg and Smith, 2009; Golombok et al., 2014, 2018; Farr, 2017; Calzo et al., 2019). Moreover, studies of LG parents (including adoptive parents specifically) have described relatively few mental health symptoms and low psychological distress, below clinical cutoffs, and often failed to uncover differences in comparison to heterosexual parents (Goldberg and Smith, 2011; Lavner et al., 2014; Calzo et al., 2019).

Despite the abundance of research on children’s and parents’ outcomes in LG parent families, we know relatively little about LG-specific family processes and comparatively less about LG *adoptive* parent families (Farr et al., 2019a; Reczek, 2020). More recently, research has increasingly emphasized unique family processes in sexual minority parent families (Golombok et al., 2014; Farr et al., 2019a, b). One factor to consider, potentially affecting adjustment and family relationships among LG adults as well as adoptive parents, is stigma. Herek (2016, p. 397), referring to Goffman’s (1963), p. 5) description of stigma as “undesired differentness” within and across social interactions, goes on to describe *sexual stigma* more specifically “to refer broadly to all facets of stigma associated with same-sex desires, sexual behaviors, and relationships, as well as sexual minority communities.”

In this paper, we also focus on *adoption stigma^1^*, which we describe as stigma based on the absence of biological ties within families and the cultural prioritizing of biological parenthood (Freeark et al., 2005; Goldberg et al., 2011; Baden, 2016; Morgan and Langrehr, 2019). From minority stress theory, we expect that marginalized groups such as LG adults as well as adoptive families may experience some negative psychological effects (i.e., stress, emotional dysregulation, social or interpersonal difficulties, rumination, etc.) resulting from stigma and discrimination (Bos et al., 2004b; Hatzenbuehler, 2009). Thus, we sought to contribute to research in these areas specifically among LG adoptive parents. In the sections that follow, we review literature relevant to individual mental health outcomes, parenting competence, as well as parent–child relationships, particularly among LG and adoptive family systems. We specifically focused on adoptive families with young children (i.e., early and middle childhood), given that these developmental periods are characterized by heightened awareness among children about different family types (i.e., based on biological and adoptive ties) and increased understanding about adoption (Brodzinsky, 2011). We incorporate specific lenses of family systems theory, and specifically, family stress theory, cultural stigma surrounding adoption, and minority stress theory.

## Family Systems Theory and Family Stress Theory

Family systems theory posits that a comprehensive understanding of individual development necessitates consideration of the family context (Minuchin, 1988; Cox and Paley, 1997; Feinberg, 2003). From a family systems perspective, processes within the family, such as relationship dynamics, are often more important to individual outcomes than is the structure of the family. Indeed, these principles are applicable to a variety of diverse family structures, including adoptive families and those headed by LG parents (Lamb, 2012; Patterson et al., 2015; Farr et al., in press). Contextual models of family stress describe how families contend with crises and why some families demonstrate better adaptation than others (Patterson, 1988; Boss et al., 2016). McCubbin and Patterson’s (1983) double ABCX model (adapted from Hill, 1949) posits that family stressors (and their pileup over time; A) interact with family coping skills through available resources (B) and perceptions and meaning-making (C) to produce outcomes in terms of family adaptation or maladaptation to the stress (or crisis; X). Family stress is influenced by a variety of internal and external factors such as place in the developmental life cycle, family structure, culture, genetics, values, and beliefs (Boss et al., 2016). While internal factors may be modifiable, external ones may be outside of families’ control. One external context that is particularly relevant for adoptive families and those headed by sexual minority parents is the role of societal and interpersonal stigma and resulting minority stress. Indeed, Prendergast and MacPhee (2018) describe a theoretical model of family resilience among LG parent families, building from minority and family stress theories, in which effects of stigma and discrimination on individual adjustment and family relationships may be buffered or exacerbated by how well families respond to these adverse experiences.

## Adoption Stigma

In the US, prevailing cultural norms about “the family” reflect heteronormativity and biological connections (i.e., bionormativity) between parents and children, as well as among siblings within families (Wegar, 2000; Fisher, 2003; Freeark et al., 2005; Baker, 2008). These “master narratives” (i.e., broad societal, cultural, and historical scripts; Hammack and Cohler, 2011) can result in stigma toward families not defined by biological ties, such as adoptive families (Miall, 1987, 1996; Baden, 2016). American women who hold greater pronatalist beliefs (e.g., valuing procreation and motherhood) may be more likely to consider adoption only after first seeking fertility treatment (Park and Wonch Hill, 2014). Indeed, willingness for some American families to adopt a child may increase after unsuccessful attempts to conceive biologically (Bausch, 2006), and infertility is often a motivator for heterosexual parent families to adopt (Farr and Patterson, 2009; Malm and Welti, 2010). As such, adoptive parents may experience grief related to their loss of not having biologically related children, which may be particularly salient during the transition to parenthood (Pinderhughes and Brodzinsky, 2019). Relatedly, many (heterosexual) adoptive parents describe feeling as if they are illegitimate, second-rate, or inferior as compared to parents with biologically related children (Miall, 1987; Wegar, 2000). Adoptive parents face stigma from others indicating that adoption is a “second-best” option for parenthood, less permanent or authentic, and that their adopted children are not their “natural” or “real” children (March, 1995; Freeark et al., 2005; Brodzinsky, 2011; Baden, 2016; Morgan and Langrehr, 2019). For example, some adoptive parents have reported that receiving family support was conditional on the biological relatedness of their child to that family member (Patterson et al., 1998).

Feelings of perceived and internalized adoption stigma have also been demonstrated among LG couple samples across their transition to adoptive parenthood and have been linked to greater depressive symptoms (Goldberg et al., 2011). Some LG couples report experiencing discrimination (Goldberg et al., 2007; Mallon, 2011; Goldberg, 2012) and additional legal complexities (e.g., living in an area without anti-discriminatory policies protecting LG adoptive parent candidates; Farr and Goldberg, 2018) when trying to adopt due to their sexual identity. Thus, the transition to parenthood is often a vulnerable time for newly formed LG adoptive parent families to face additional experiences of stigma because of the already heightened levels of stress and depressive symptoms that adoptive parents may feel when becoming parents (McKay et al., 2010; Goldberg and Smith, 2011). Indeed, LG adoptive parents face stigma not only on the basis of adoption but also on the basis of sexual orientation. It is to this topic of minority stress resulting from sexual stigma that we turn to next.

## Minority Stress Theory and Lesbian and Gay Adoptive Parent Family Outcomes

Meyer’s (2003) minority stress theory is based on the premise that sexual minority individuals experience often chronic levels of psychosocial stress resulting from stigma, prejudice, and discrimination. Minority stress can specifically result from microaggressions, defined as somewhat subtle or more covert slights or insults (Sue et al., 2007). Minority stress resulting from more overt discrimination as well as from microaggression experiences is associated with negative health outcomes among sexual minority adults (Wright and Wegner, 2012; Nadal, 2013; Wegner and Wright, 2016). LG individuals are often affected by homophobic microaggressions—those that are based on sexual minority group membership (e.g., overhearing derogatory epithets like “that is so gay” or assumptions that one’s sexual orientation is heterosexual; Wright and Wegner, 2012; Nadal, 2013, 2019). Such microaggressions can also be specifically directed toward same-gender parent families (e.g., asking a child with two gay fathers where their “mother” is; Farr et al., 2016a). Stigma and prejudice directed toward LG individuals may also operate differently for men and women (Herek, 2009). For example, gay men often face additional unique barriers when attempting to become parents (e.g., inability to bear children; Goldberg, 2012) and scrutiny related to their parenthood (Tornello and Patterson, 2015; Carneiro et al., 2017)—which may in part be attributable to cultural stigma and negative attitudes toward fatherhood (McCutcheon and Morrison, 2015). Thus, it is important to consider how intersecting identities such as gender and sexual orientation may affect experiences of stigma and homophobic microaggressions in parents. Although research regarding microaggressions experienced by sexual and gender minority persons is advancing (Fisher et al., 2019; Nadal, 2019), homophobic microaggressions and their possible associations with individual and family outcomes have not been specifically examined (to our knowledge) among a sample of LG parents, let alone LG adoptive parents. Thus, research in this area would provide greater understanding about the potentially unique experiences of LG adoptive parent families and how to support healthy and successful adoptive placements in the context of minority stress.

What we know from existing research is that children and their (adoptive) sexual minority parents do face stigma, overt discrimination, and microaggressions based on parental sexual orientation (Bos and Gartrell, 2010; Vyncke et al., 2014; Farr et al., 2016a; Haines et al., 2018; Green et al., 2019). Moreover, these minority stress experiences have been associated with a variety of negative outcomes among sexual minority parent family members, such as lower behavioral adjustment, negative health outcomes, lower well-being, and less positive parenting and coparenting (Tornello et al., 2011; Lick et al., 2013; Crouch et al., 2014, 2015; Carone et al., 2017, 2018; Golombok et al., 2018; Calzo et al., 2019; Goldberg et al., 2019; Green et al., 2019). In terms of understanding associations between individual adjustment and homonegative microaggressions in particular, research has demonstrated that it is important to include consideration of past and current experiences, as well as perceptions of their impact (and how this interacts with past or current experiences; Wright and Wegner, 2012).

Moreover, LG parents may encounter additional or particularly salient experiences of stigma related to their parenting ability and sexual orientation during their transition to parenthood (e.g., discrimination from adoption agency workers; Mallon, 2011). Indeed, examining the presence and perceived impact of past and current homonegative microaggressions is important in understanding the contextual factors that may influence parent adjustment and family relationships. Connecting with family stress theory, some scholarship in this area has highlighted how the negative consequences of minority stress are often a product of broader familial stress resulting from stigma rather than, or in addition to, overt individual experiences (Crouch et al., 2017; Prendergast and MacPhee, 2018). Thus, from minority and family stress perspectives, we sought to examine how stigma related to adoption and sexual orientation might be differentially associated with mental health and perceptions of parenting competence among LG and adoptive parents, as well as with children’s reports of parent–child relationship quality.

## The Current Study

Among a sample of approximately 100 adoptive families headed by lesbian, gay, and heterosexual parents, we explored associations at two points (about 5 years apart; when children were preschool-age and school-age, respectively) among parent mental health symptoms, perceived parenting competence, perceived adoption stigma, homonegative microaggressions, and quality of parent–child relationships. We also examined whether any of these variables of interest differed as a function of parental sexual orientation and parent gender identity (i.e., lesbian women, gay men, heterosexual women, and heterosexual men), as well as family type (i.e., those headed by lesbian mothers, gay fathers, and heterosexual parents).

## Aims, Research Questions, and Hypotheses

1. The first aim was to examine possible differences in variables of interest as a function of parent gender and sexual identity as well as family type (i.e., lesbian, gay, or heterosexual parent families). Would differences emerge in mental health symptoms, perceived parenting competence, or adoption stigma as functions of parent gender and/or sexual identity? Given distinct experiences of stigma between lesbian mothers and gay fathers (Herek, 2009; Goldberg, 2012; Tornello and Patterson, 2015; Carneiro et al., 2017), would there be differences as a function of parent gender in homonegative microaggression experiences? Finally, would there be differences by family type in children’s perceptions of parent–child relationship quality? We generally anticipated few differences as a function of family type but queried whether we might find differences based on parent gender in parenting competence, given previous literature (Freeark et al., 2005; Goldberg and Smith, 2009; Calzo et al., 2019). In contrast, we also considered a competing hypothesis based on family stress and minority stress theories. Related to possible pileup effects of stress (McCubbin and Patterson, 1983) resulting from both adoption and sexual stigma, we explored whether outcomes in our variables of interest among LG adoptive parent families might be distinct from those among heterosexual adoptive parent families.
2. The second aim was to investigate associations across time among parent mental health symptoms and perceived parenting competence, both assessed when children were in preschool, with experiences of adoption stigma, homonegative microaggressions, and parent–child relationship quality, all evaluated 5 years later. Given some previous research examining similar linkages between mental health, parenting, and adoptive family relationships (Goldberg and Smith, 2009; Brodzinsky, 2011; Goldberg et al., 2011), we hypothesized that greater mental health symptoms and lower perceived parenting competence would be linked to perceptions of greater adoption stigma and lower relationship quality, respectively. Based on our theoretical frameworks of family and minority stress as well as some relevant existing research regarding sexual stigma and homonegative microaggressions as related to LG individual and parent outcomes (Goldberg et al., 2011, 2019; Tornello et al., 2011; Wright and Wegner, 2012; Carone et al., 2017; Green et al., 2019), we also anticipated that greater mental health symptoms and lower competence, respectively, would be associated with more microaggressions.
3. The third and final aim was to investigate whether stigma and microaggressions would be concurrently associated with parent–child relationship quality, all assessed during middle childhood. Based on existing research on parallel constructs (e.g., Goldberg et al., 2011), and building from minority stress and family stress theories, we predicted that adoption stigma described by parents would predict children’s reports of lower parent–child relationship quality (accounting for parent mental health and perceived competence). Aligned with some research indicating associations between greater sexual stigma, family stress, and child outcomes (Bos and Gartrell, 2010; Vyncke et al., 2014; Crouch et al., 2017; Carone et al., 2018; Calzo et al., 2019), we also expected that homonegative microaggressions experienced by LG parents would predict reports of lower parent–child relationship quality (accounting for parent mental health symptoms, competence, and adoption stigma) among their children.

## Materials and Methods

### Participants

Data presented here are from the first (W1) and second (W2) waves of an ongoing longitudinal study examining lesbian, gay, and heterosexual parent adoptive families in the US (Farr, 2017). Parents in this study were recruited for W1 from five private adoption agencies across the US that offered options for domestic infant adoptive placements. These agencies were in areas where LG couples could legally adopt in the mid-2000s. Parents were eligible to participate if they had completed a private domestic infant adoption. A total of 106 two-parent families (27 lesbian, 29 gay, 50 heterosexual couples) and their eldest adopted child (in the age range of 1–5 years old; i.e., the target child) participated at W1. In W2, 96 families participated (26 lesbian, 29 gay, 41 heterosexual couples) in some capacity. Not all participants, however, fully completed every measure at each time point (see section “Measures” below for more details about missingness). The retention rate between W1 and W2 for this sample was 90.6% (26 lesbian, 29 gay, 41 heterosexual parent families). Families lived across the US (but predominantly the US South, East Coast, and West Coast), and most participants (74.5%) lived in an urban (versus rural) area as defined by US Census population sizes; there were no changes in geographic regions among participating families from W1 to W2.

Of the families represented in the measures used in this paper at W2, almost half (45.3%) of the children were transracially adopted, with children being more racially diverse than their parents. Most children were described by their parents as white/Caucasian (37.8%), followed by Black/African American (31.1%), Multi-Ethnic/Multi-Racial (25.6%), Latino/Hispanic (3.3%), Asian American (1.1%), and Native American/American Indian (1.1%). Parents self-reported their racial/ethnic identities, and most identified as white/Caucasian (84.8%), followed by Black/African American (10.7%), Latino/Hispanic (1.7%), Multi-Ethnic/Multi-Racial (1.1%), Other (1.1%), and Asian American (0.6%). Gender was almost equally split among children (52.2% female) and parents (48.3% female); all identified as cisgender. At the time of data collection during W2, children were 8.36 years of age on average (*SD* = 1.66), and parents were about 47.56 years old (*SD* = 5.87). Parents had a median annual total household income of $160,000 (*SD* = 110,976) and were well-educated with 89.2% holding at least a college degree. Additional participant demographic information from W2 can be found in Table 1 (see Farr, 2017 for sample demographics at W1).

**TABLE 1:** Demographic information wave 2 (W2) by family type.

	*N* = 96 families			
Variable	Lesbian parents	Gay parents	Heterosexual parents	Sample
**Family**				
Household income ($K)^a^	146 (129)	192 (107)	150 (86.76)	160 (111)
Transracial adoptions	48%	58.6%	34.1%	45.3%
**Parents**				
Age (years)	48.51 (5.01)	46.85 (6.06)	47.48 (6.18)	47.56 (5.87)
Race (% white)	84.4%	83%	86.3%	84.8%
Education (% at least college degree)	97.7%	88.5%	82.7%	89.2%
Work status (% full-time)	75%	75%	63.5%	70.9%
**Children**				
Gender (% female)	65.2%	40.7%	52.5%	52.2%
Age (years)	8.48 (1.73)	8.26 (1.51)	8.35 (1.76)	8.36 (1.66)
Race (% white)	39.1%	29.6%	42.5%	37.8%

*^*a*^Median annual income. *SD*s are given in parentheses. W2 = wave 2. Aside from household income, *F*(2,85) = 6.61, *p* < 0.01, there were no significant differences by family type in any of these demographic variables. Demographic information for this sample at W2 was also originally reported in Farr, 2017.*

### Procedure

To recruit participants for W1, researchers collaborated with five domestic private infant adoption agencies in the US mentioned previously. Agency directors then forwarded a study invite to families with whom children had been placed recently or within the past few years. Interested participants contacted the research team, and the first author conducted 2-h home visits with each participating family (*N* = 106) to collect observational and survey data (e.g., Farr et al., 2019a). Both parents individually completed a demographic questionnaire and other measures *via* paper-and-pen surveys during the visit.

Participants in W1 were recontacted by the research team about 5 years later and invited to participate in W2. Some measures below were administered only during W2, and some were administered in both waves—all were self-report. Questionnaires at W2 were administered via the online survey platform Qualtrics. Parents independently completed surveys at their leisure. Children were assisted with completing the child-level questionnaire [i.e., the Inventory of Parent and Peer Attachment (IPPA), described below] by the first author during a scheduled home visit. Participants were not compensated, and participation was voluntary. Informed consent was provided by parents for their own and their children’s participation; assent was obtained from children. All study materials and procedures were approved by the Institutional Review Boards of the University of Virginia, the University of Massachusetts Amherst, and the University of Kentucky. Data were collected between 2007–2009 (W1) and 2013–2014 (W2).

### Measures

#### Demographic Characteristics

Both parents individually completed questionnaires related to their and their children’s demographic information at both waves. Parents were asked about their racial/ethnic background and the racial/ethnic background of the target child. Transracial adoption in this sample was defined as the target child’s race being different than at least one of the parents—this operationalization of transracial adoption has been used in other studies (Zhang and Lee, 2011; Jacobson et al., 2012; Marr, 2017). Parents were also asked for their date of birth and that of the target child to assess their age at the time of data collection during both waves. Child and parent gender, total household income, parent education status, and parent sexual orientation were also assessed.

In W1, parents were provided with the options of “straight/heterosexual,” “lesbian,” “gay,” “bisexual,” or “questioning/uncertain” and asked to select the one that best represented their sexual orientation. In W2, parents were provided with an additional “other/self-describe” write-in option. In W1, eight of the mothers in female-partnered couples and two of the mothers with male partners identified as bisexual. One male parent with a female partner identified as bisexual in W1. In W2, five of the mothers in the female-partnered couples identified as bisexual, and two mothers in the female-partnered couples self-identified as queer. One male parent with a male partner identified as questioning/uncertain. Given the small cell sizes in our analyses, we include individuals in different-gender couples as heterosexual and participants in same-gender couples as lesbian or gay—a method used in other studies examining sexual minority and heterosexual adoptive parents (e.g., Brodzinsky and Goldberg, 2016; Wyman Battalen et al., 2019). This collapsing of individual sexual minority identities (e.g., bisexual) into broader groups (e.g., lesbian) may contribute to identity erasure (e.g., bi-erasure; Hackl et al., 2013) as it is inconsistent with how participants self-identify. This generalized categorization may also overlook variability across individual identities (Brodzinsky and Goldberg, 2016). Despite these limitations, we utilize this method of classifying participants to preserve power for our analyses.

#### Mental Health Symptoms

To assess the presence of mental health symptoms and psychological distress, parents completed the Brief Symptom Inventory (BSI; Derogatis and Melisaratos, 1983) at both W1 and W2. This widely used clinical measurement survey contains 53 items across nine domains each with corresponding subscales: depression, anxiety, somatization, obsession–compulsion, interpersonal sensitivity, hostility, phobic anxiety, paranoid ideation, and psychoticism. Participants were asked, “*In the past 7 days, how much were you distressed by?*” and then presented with the list of items (e.g., *Feeling hopeless about the future*). Items are scored on a Likert scale ranging from 0 (*not at all*) to 4 (*extremely*). All 53 items^2^ were summed and averaged to create a Global Severity Index (GSI)—higher scores indicate higher levels of overall psychological distress. In W1, 208 parents (four one-parent reports) completed this measure and had a Cronbach’s alpha (α) of 0.94. At W2, 175 parents completed this measure (α = 0.92). We note that high α values (e.g., α > 0.90) can result from alpha inflation from the large number of items (Streiner, 2003; Tavakol and Dennick, 2011).

#### Parenting Competence

The childcare competence subscale from the Who Does What? Measure (WDW-C; Cowan and Cowan, 1990) was completed by parents at both W1 and W2 to assess their perceived competence in parenting the target child. There are 20 items (e.g., *Disciplining our child*) on a Likert scale ranging from 1 (*not at all competent*) to 5 (*very competent*). All 20 items are summed and averaged to create a total competence score. Higher scores indicate higher perceived parenting competence. In W1, 210 parents completed this measure (α = 0.91). At W2, 171 completed this measure (α = 0.92).

#### Adoption Stigma

The Feelings About Adoption Scale (FAAS; Goldberg et al., 2011) was used to measure how aware adoptive parents are about adoption stigma (*perceived stigma subscale*) and if they internalize this stigma (*internalized stigma subscale*). The internalized stigma subscale had low reliability (α = 0.47) in the scale validation analysis (Goldberg et al., 2011) and in our sample (α = 0.17). Thus, we only used the perceived stigma subscale (sample α = 0.81). This subscale contains five items assessing participants’ perceptions of adoptive stigma (e.g., *People in society value biological ties over everything else in creating a family*). This scale was only administered in W2 with 177 parents completing the scale.

#### Homonegative Microaggressions

Only LG parents (*n* = 94) completed the Homonegative Microaggressions Scale (HMS; Wright and Wegner, 2012), which contains 45 items assessing experiences of homonegative microaggressions (e.g., *How often have people conveyed that it is your choice to be gay?*). The scale was validated in individuals identifying as cisgender and lesbian, gay, or bisexual. There are three subscales (past, current, and impact); each asks for a rating on all 45 items. The past subscale (HMS-P; α = 0.92) asks participants to think about their experiences growing up, the current subscale (HMS-C; α = 0.88) asks about the last 6 months, and the impact subscale (HMS-I; α = 0.96) asks participants to rate how much the event bothered or impacted them. The items are scored on a Likert scale ranging from 1 (*hardly ever/never/not at all*) to 5 (*constantly/a great deal*), and there is an option for participants to indicate if the question is not applicable to them. Means were calculated for each subscale. Higher scores indicate more frequent experiences or greater impact. Additionally, as recommended by Wright and Wegner (2012) for the HMS scale, interaction variables were created for past and impact subscale scores (HMS-PI) as well as current and impact subscale scores (HMS-CI). In the scale validation study, experiencing a past homonegative microaggression was significantly moderated by impact in predicting self-esteem (Wright and Wegner, 2012). Individuals who experienced greater past homonegative microaggressions were more likely to report having lower self-esteem when those experiences were highly impactful for the participant. As such, these interaction terms (i.e., HMS-PI and HMS-CI) were included in all analyses using this measure. This measure was only administered during W2.

#### Parent–Child Relationship Quality

Children (*n* = 90) completed the IPPA (Armsden and Greenberg, 1987) at W2 only. The IPPA assesses children’s feelings of closeness and overall relationship quality with their parents (e.g., *I feel my parent does a good job as my parent).* Children completed one report for each parent (28 items each; α = 0.85^3^). The IPPA consists of three subscales: trust, communication, and alienation. We created a composite score that provides a mean of all items, averaged across both parents. Higher scores indicate better relationship quality.

### Data Analytic Plan

Hierarchical linear modeling (HLM) was used (with HLM7 software; Raudenbush et al., 2011) to account for shared variance and interdependent responses within families (often two parents reporting from the same family or children reporting on their two parents within families) for dependent variables of interest (Raudenbush and Bryk, 2002). First, we examined unconditional models with no predictors and only the outcome variables of interest (i.e., mental health symptoms, parenting competence, adoption stigma, homonegative microaggressions, parent–child relationship quality). HLM is warranted only when intraclass correlation coefficients (ICCs) exceed the cutoff value of 25% (Guo, 2005). ICCs were below this cutoff for outcome variables of parent mental health symptoms, perceived parenting competence, perceived adoption stigma, and homonegative microaggressions, but HLM was warranted for parent–child relationship quality with an ICC of 58%. The basic equations for the HLM models are: Level 1: *Y*_ij_ = β_0__j_ + *e*_ij_ and Level 2: β_0_*_j_* = γ*_00_* + *u_0__j_*. Level 1 represents the calculation for parent–child relationship quality, *Y*_ij_. β_0_*_j_* represents the random intercept, and *e*_ij_ represents the error term. Level 2 represents a comparison of averages for the outcome variable. Interdependence of responses within families is controlled by the *u_0__j_* coefficient.

### Missing Data

As recommended, we examined the data for possible patterns of missingness to explain non-participation (Acock, 2005; Widaman, 2006; Jeličić et al., 2009; Johnson and Young, 2011). Missingness in terms of item non-response on key variables (mental health symptoms, perceived parenting competence, perceived adoption stigma, parent–child relationship quality, all five homonegative microaggression variables) was low for W1 variables (averaging 1.4%) and moderate (between 10 and 20%; Widaman, 2006) for W2 variables (averaging 17%). To account for missingness, we used full information maximum likelihood (FIML) in the HLM models, an approach that is both widely recommended and appropriate for managing missing data in multilevel models (Acock, 2005; Widaman, 2006; Johnson and Young, 2011). We made use of listwise deletion for other analyses; this “traditional” technique has been demonstrated as robust when predictor variables show low missingness and as related to the type of missingness that frequently characterizes data in studies of families (Jeličić et al., 2009; Johnson and Young, 2011).

### Power Analyses

Power analyses were conducted using G^∗^Power (Faul et al., 2007) for analyses of interest with alpha set to α = 0.05 with the sample size of *N* = 96 families represented at W2. For bivariate correlations among variables of interest, achieved power was 0.99 for large, 0.85 for medium, and 0.16 for small effects. For one-way ANOVA with four groups (lesbian mothers, gay fathers, heterosexual mothers, heterosexual fathers), achieved power was 0.91 for large, 0.50 for medium, and 0.11 for small effects. For multiple regression (three predictors), achieved power was 0.99 for large, 0.89 for medium, and 0.18 for small effects. Thus, analyses were mostly powered to detect medium to large effects.

## Results

### Preliminary Analyses

Preliminary analyses (i.e., bivariate Pearson two-tailed correlations) were run to assess the presence of significant associations between all variables of interest (Table 2). Preliminary analyses were also conducted to explore the role of possible covariates in analyses for all variables of interest (parent mental health symptoms, perceived parenting competence, perceived adoption stigma, homonegative microaggression experiences, and children’s perceptions of parent–child relationship quality). Given previous research indicating the relevance to parent adjustment and family relationships of each of the following variables—child age (Farr, 2017), child gender (Freeark et al., 2005), presence of siblings (Farr et al., 2016b), birth/age order of children (Barth and Brooks, 1997), parent socioeconomic status (e.g., income, education; Neiss and Rowe, 2000; Johnson et al., 2007), geographic location (i.e., urbanicity; Kinkler and Goldberg, 2011), and transracial adoption status (Baden, 2016) among samples of adoptive families (including those with LG parents)—we considered all as possible covariates. As we conducted a series of dependent variables and demographic covariates, we applied a Bonferroni correction (α = 0.01). These analyses revealed no significant associations among covariates and variables of interest, so no demographic variables were included in subsequent analyses.

**TABLE 2:** Preliminary correlations for all variables of interest.

Variable	1	2	3	4	5	6	7	8	9	10
1. Parent–child relationships	–									
2. Mental health (W1)	–0.04	–								
3. Mental health	0.04	0.52***	–							
4. Competence (W1)	0.11	−0.33***	−0.26***	–						
5. Competence	0.27***	−0.28***	−0.54**	0.57***	–					
6. Adoption stigma	0.08	0.16*	0.08	–0.02	–0.12	–				
7. Past homonegative microaggressions	–0.08	0.09	0.28**	– 0.02	–0.13	0.23*	–			
8. Current homonegative microaggressions	–0.05	0.14	0.18	0.01	–0.04	0.35**	0.58***	–		
9. Impact of homonegative microaggressions	–0.02	0.13	0.19	–0.07	–0.04	0.45***	0.57***	0.51***	–	
10. Past*impact homonegative microaggressions	–0.07	0.12	0.23*	–0.04	–0.10	0.38***	0.82***	0.63***	0.91***	–
11. Current*impact homonegative microaggressions	–0.02	0.15	0.16	0.001	–0.02	0.41***	0.60***	0.86***	0.83***	0.86***

*p < 0.05. **p < 0.01. ***p < 0.001. W1 = wave 1. All other measures administered at wave 2.

### Descriptive Results and Group Differences

A one-way ANOVA was conducted to assess differences by parent gender and sexual identity (four groups; lesbian mothers, gay fathers, heterosexual mothers, heterosexual fathers) in mental health symptoms, perceived parenting competence, and adoption stigma (Table 3). No significant differences were found by parent gender or sexual identity in mental health symptoms at W1 (child *M*_age_ = 3.01) or W2 (child *M*_age_ = 8.36). Significant differences were found, however, in perceived parenting competence at W1 and W2. A Tukey *post hoc* analysis revealed that heterosexual fathers were significantly different at W1 from lesbian mothers (*p* < 0.001), gay fathers (*p* = 0.001), and heterosexual mothers (*p* < 0.001). At W2, heterosexual fathers were also significantly different from lesbian mothers (*p* < 0.001), gay fathers (*p* < 0.001), and heterosexual mothers (*p* < 0.001). In both waves, heterosexual fathers reported feeling less competent in their parenting ability than all other groups (see Table 3 for descriptive information). No significant differences were found among the remaining three groups for perceived parenting competence. Finally, no significant differences were found in perceived adoption stigma at W2 by parent gender or sexual identity.

**TABLE 3:** Means, standard deviations, and ANOVA by family type and parent gender.

	Lesbian mothers	Gay fathers	Heterosexual mothers	Heterosexual fathers	Heterosexual parents	Total			
Variable	*M (SD)*	*M (SD)*	*M (SD)*	*M (SD)*	*M (SD)*	*M (SD)*	*F(df)*	*p*	η^2^
	*n* = 45	*n* = 54	*n* = 40	*n* = 40	*n* = 80	*n* = 179			
Parent–child	4.56 (0.40)	4.33 (0.52)	4.48 (0.43)	4.50 (0.52)	4.49 (0.47)	4.46 (0.47)	–	–	–
relationships^a^	*n* = 52	*n* = 58	*n* = 50	*n* = 49	*n* = 99	*N* = 209			
Mental health	0.58 (0.34)	0.57 (0.41)	0.54 (0.34)	0.55 (0.38)	0.54 (0.36)	0.56 (0.37)	0.14(3,208)	0.936	0.002
(W1)	*n* = 43	*n* = 52	*n* = 41	*n* = 39	*n* = 80	*N* = 175			
Mental health	0.29 (0.24)	0.30 (0.28)	0.23 (0.15)	0.34 (0.29)	0.29 (0.24)	0.29 (0.25)	1.30(3,171)	0.275	0.02
	*n* = 54	*n* = 58	*n* = 48	*n* = 49	*n* = 97	*N* = 209			
Competence	4.68 (0.30)	4.60 (0.43)	4.78 (0.27)	4.29 (0.61)	4.53 (0.53)	4.59 (0.46)	12.25(3,205)	<0.001	0.15
(W1)	*n* = 41	*n* = 52	*n* = 39	*n* = 39	*n* = 78	*N* = 171			
Competence	4.69 (0.34)	4.65 (0.36)	4.70 (0.34)	4.24 (0.63)	4.47 (0.56)	4.58 (0.46)	10.17(3,167)	<0.001	0.15
	*n* = 46	*n* = 52	*n* = 40	*n* = 39	*n* = 79	*N* = 177			
Adoption stigma	2.24 (0.92)	2.06 (0.73)	2.34 (0.94)	2.22 (0.81)	0.28 (0.88)	2.21 (0.85)	0.85(3,173)	0.470	0.01

*The four groups included in the ANOVA analyses were lesbian mothers, gay fathers, heterosexual mothers, and heterosexual fathers. A Tukey *post hoc* test revealed that heterosexual fathers had significantly lower scores than all other groups in competence (W1) and competence. W1 = wave 1. All other measures administered at wave 2. ^*a*^This variable was assessed *via* hierarchical linear modeling (HLM) rather than ANOVA. Descriptive information presented here only.*

Five separate independent samples *t*-tests were conducted to assess differences between LG parents on the five homonegative microaggression variables at W2. No significant differences were found between LG parents among any of the five homonegative microaggression variables (Table 4). HLM was used to assess differences by family type (three groups: lesbian, gay, and heterosexual parent families) in child-reported scores of parent–child relationships. Specifically, to compare by family type, the Level 2 equation provides a comparison of averages across family type, e.g., Level 2: β*_0__j_* = γ*_00_* + γ*_01_*(*Lesbian*) + γ*_01_*(*Gay*) + *u_0__j_*. As in previous HLM research involving indistinguishable dyads (e.g., same-gender couples; Smith et al., 2013), the Level 2 coefficients reflect the effects of being “lesbian versus heterosexual” and “gay versus heterosexual” on parent–child relationship quality. No significant differences were found in this variable among lesbian, gay, or heterosexual parent families. We also conducted these same analyses a second time with gay father families as the reference group such that comparisons were directly made between gay father families and lesbian mother families and between gay father families and heterosexual parent families. The pattern of results was the same regardless of whether lesbian or gay parent families were the reference group.

**TABLE 4:** Homonegative microaggressions: means, standard deviations, *t*-tests, and effect sizes for lesbian mothers and gay fathers.

	Lesbian mothers (*n* = 43)	Gay fathers (*n* = 51)	Total (*n* = 94)			
Variable	*M* (*SD*)	*M* (*SD*)	*M* (*SD)*	*t*(df)	*p*	*d*
Past homonegative microaggressions	2.24 (0.71)	2.48 (0.73)	2.37 (0.73)	−1.6 (92)	0.935	0.33
Current homonegative microaggressions	1.67 (0.61)	1.64 (0.41)	1.65 (0.50)	0.30 (92)	0.155	0.06
Impact of homonegative microaggressions	2.12 (0.96)	2.16 (0.82)	2.14 (0.88)	−0.19 (92)	0.871	0.04
Current*impact Homonegative microaggressions	3.82 (3.47)	3.71 (2.20)	3.76 (2.83)	0.19 (92)	0.605	0.04
Past*impact homonegative microaggressions	5.11 (3.43)	5.72 (3.43)	5.44 (3.65)	−0.81 (92)	0.736	0.18

*Asterisks refers to interaction terms.*

### Associations Across Wave 1 and Wave 2

First, paired samples *t*-tests were conducted to assess differences between W1 and W2 for parents’ mental health symptoms and perceived parenting competence. A significant difference was found between W1 and W2 for mental health symptoms, *t*(172) = 11.73, *p* < 0.001; parents’ mental health symptom scores were significantly higher at W1 than W2 (see Table 3 for descriptive information). No significant difference was found, however, between W1 and W2 means for perceived parenting competence. Next, we regressed adoption stigma at W2 onto parents’ feelings of parenting competence and their mental health symptoms at W1. The omnibus model was not significant so we did not interpret the individual predictors (see Supplementary Material). We also used HLM to see if parents’ mental health symptoms and feelings of parenting competence at W1 predicted child-reported parent–child relationship quality at W2. Neither parents’ mental health symptoms nor feelings of parenting competence at W1 significantly predicted parent–child relationship quality. Finally, we regressed all five homonegative microaggression variables at W2 onto parents’ mental health symptoms and feelings of parenting competence at W1 for LG parents. Given the large number of statistical tests, we applied a Bonferroni correction with alpha set to *p* = 0.01. All five omnibus models were not significant, so we did not interpret the individual predictors (see Supplementary Material).

### Cross-Sectional Associations Within Wave 2

For our research questions pertaining to the entire sample, we regressed adoption stigma at W2 onto parents’ mental health symptoms and perceived parenting competence at W2. The omnibus model was not significant, so we did not interpret the individual predictors (see Supplementary Material). Using HLM, results indicated that only perceived parenting competence was a significant predictor of parent–child relationship quality—parents’ mental health symptoms and adoption stigma were not significant predictors of parent–child relationship quality (Table 5).

**TABLE 5:** Hierarchical linear modeling (HLM): inventory of parent and peer attachment from wave 2 (W2) variables (whole sample).

Variable	Coefficient	*SE*	*t*	*df*	*p*
Intercept β*_0_*					
Intercept γ*_00_*	4.45	0.04	102.59	86	<0.001
Competence β*_1_*					
Intercept γ*_10_*	0.25	0.07	3.79	72	<0.001
Mental health β*_2_*					
Intercept γ*_20_*	0.16	0.13	1.27	72	0.208
Adoption stigma β*_3_*					
Intercept γ*_30_*	0.02	0.04	0.45	72	0.652
**Random effect**	***SD***	**Variance**	***df***	**χ*^2^***	***p***
Intercept, *u*_0_	0.36	0.11	86	280.67	<0.001
level1, *r*	0.31	0.09			

For our research questions pertaining to LG parent families, we regressed the five individual homonegative microaggression variables at W2 onto parents’ mental health symptoms and perceived parenting competence at W2. None of the five homonegative microaggression variables were significantly predicted by parents’ mental health symptoms or perceived parenting competence at W2 (i.e., only one omnibus model was significant with a Bonferroni correction applied, with greater W2 mental health symptoms statistically predicting greater past homonegative microaggressions; see Supplementary Material). We then included all five homonegative microaggression variables, parents’ mental health symptoms, perceived parenting competence, and adoption stigma in an HLM model to assess if any of those W2 variables predicted parent–child relationship quality at W2 (Table 6). Only current experiences (i.e., within the last 6 months) of homonegative microaggressions significantly predicted parent–child relationship quality such that when parents experienced more microaggressions, child-reported parent–child relationship quality was lower.

**TABLE 6:** Hierarchical linear modeling (HLM): inventory of parent and peer attachment from wave 2 (W2) variables in lesbian and gay (LG) parent families.

Variable	Coefficient	*SE*	*t*	*df*	*p*
Intercept β*_0_*					
Intercept γ*_00_*	4.41	0.06	71.19	46	<0.001
Competence β*_1_*					
Intercept γ*_10_*	0.17	0.14	1.20	30	0.238
Mental health β*_2_*					
Intercept γ*_20_*	0.13	0.19	0.68	30	0.499
Adoption stigma β*_3_*					
Intercept γ*_30_*	0.05	0.06	0.84	30	0.410
Current homonegative microaggressions β*_4_*					
Intercept γ*_40_*	−0.68	0.31	−2.21	30	0.035
Past homonegative microaggressions β*_5_*					
Intercept γ*_50_*	0.32	0.19	1.71	30	0.097
Impact of homonegative microaggressions β*_6_*					
Intercept γ*_60_*	0.07	0.19	0.38	30	0.707
Past*impact homonegative microaggressions β*_7_*					
Intercept γ*_70_*	−0.11	0.07	−1.54	30	0.133
Current*impact homonegative microaggressions β*_8_*					
Intercept γ*_80_*	0.16	0.08	1.97	30	0.058
**Random effect**	***SD***	**Variance**	**df**	**χ^2^**	**p**
Intercept, *u*_0_	0.35	0.13	46	191.36	<0.001
level-1, *r*	0.27	0.07			

## Discussion

In this study, findings revealed a generally high-functioning sample of adoptive families headed by lesbian, gay, and heterosexual parents of school-age children, with few differences uncovered as a function of parents’ gender and sexual identities. First, parents were well-adjusted overall in terms of mental health and in reporting generally high levels of parenting competence. Parents also reported relatively low adoption stigma and children described high-quality parent–child relationships on average. LG parents also described few homonegative microaggressions overall. Aligned with general predictors from both family stress (i.e., pileup effects) and minority stress theories (Patterson, 1988; Meyer, 2003), however, we did uncover several significant associations between stigma experiences and family dynamics. While LG parents in this sample did not appear to face greater mental health challenges than did heterosexual parents, current homonegative microaggression experiences were significantly connected with children’s perceptions of lower parent–child relationship quality. From a strengths-based perspective, greater parenting competence was linked with better parent–child relationship quality for all in the sample (on average), and LG parents described themselves as particularly competent in their parenting roles. In this way, our findings did not suggest any additional vulnerabilities for LG adoptive parents as compared to heterosexual adoptive parents in terms of mental health, parenting competence, or parent–child relationships, as might have been expected from family and minority stress theories; rather, our study pointed to possibly unique dynamics of resilience among these families. Our study may be the first to reveal parenting competence as a distinct strength among LG adoptive parents, aligned with family resilience theories among same-gender parent families (Prendergast and MacPhee, 2018), especially in sharing associations with children’s perceptions of closeness with their parents and despite experiences of stigma.

Our first hypothesis regarding differences as a function of parental sexual orientation and gender was generally supported. There were no significant differences in this regard in parent-reported mental health symptoms at either time point (W1, W2), supporting earlier research with LG parents (Calzo et al., 2019), LG adoptive parents specifically (Goldberg and Smith, 2011; Calzo et al., 2019; Goldberg et al., 2019), and across two time points (Lavner et al., 2014). Although there were no differences by parents’ sexual and gender identities in mental health symptoms at W1 and W2, all parents described fewer average symptoms when their children were in middle childhood as compared to 5 years earlier during early childhood. This may reflect the particularly demanding responsibilities of parenting young children (Goldberg and Smith, 2009, 2011; Lavner et al., 2014), especially considering that for many families in this sample, the target children represented the parents’ first child. There were no differences as a function of parent sexual and gender identity in reports of perceived adoption stigma, consistent with Goldberg et al.’s (2011) study of LG and heterosexual adoptive parents. Finally, there were also no differences between LG parents in their reports of homonegative microaggression experiences, which is aligned with earlier work, at least among LG individuals without children (Wright and Wegner, 2012). It is important, however, to consider that gay fathers may experience additional stigma related to the intersection of their gender and sexual identity during the transition to parenthood when compared to lesbian mothers given the cultural importance placed on motherhood and general devaluation of fatherhood (e.g., McCutcheon and Morrison, 2015; Tornello and Patterson, 2015; Carneiro et al., 2017). Future research is warranted to further explore the intersections of gender and sexual identity-related stigma and parenting.

Children also did not differ as a function of family type (lesbian, gay, or heterosexual parents) in their reports of parent–child relationship quality when they were in middle childhood (W2); children generally described high-quality relationships with their adoptive parents. Our finding aligns with the broader literature on child outcomes, parenting, and family relationships among LG parent families indicating healthy and close parent–child relationships with no differences as compared to heterosexual parent families, further underscoring the greater significance of family processes over family structure to individual and family adjustment (Erich et al., 2009b; Golombok et al., 2014, 2018; Carone et al., 2018; McConnachie et al., 2019). Previous studies, however, have generally assessed parent–child relationships from parents’ perspectives or via video-recorded observations of parent–child interaction (with the exception of McConnachie et al.’s interview-based study with children in middle childhood—average age of 11 years). Erich et al. (2009a, b) did use the same assessment tool among a sample of adolescent children adopted by lesbian, gay, and heterosexual parents. To our knowledge, however, ours is the first study to include a quantitative, self-reported assessment of the perspectives of adopted preadolescent children with LG parents about their parent–child relationships. As such, these findings represent contributions to literatures about both sexual minority and adoptive parent families.

The only significant group difference uncovered in variables of interest was with regard to parenting competence, as expected. Heterosexual fathers rated themselves as significantly less competent than the three other groups of parents (lesbian mothers, gay fathers, and heterosexual mothers) at both time points (i.e., when their children were in early and middle childhood, respectively). The broader family literature, which has largely examined the parenting experiences of heterosexual adults and sometimes as adoptive parents, has also demonstrated differences (that often reflect differential caregiving experiences) between mothers and fathers in perceived competence (e.g., Freeark et al., 2005; Lamb, 2012). In addition, in our sample, there were no significant differences in parenting competence when children were in early or middle childhood; all parents on average felt relatively competent at both time points. The generally high levels of competence may reflect that our sample is comprised of adoptive parents who undergo a rigorous screening process to evaluate their potential to be effective parents (Pinderhughes and Brodzinsky, 2019).

Related to parenting competence and sexual orientation, some earlier research comparing lesbian and heterosexual (non-adoptive) mothers has similarly demonstrated relatively high levels of parenting competence with no differences based on mothers’ sexual identities (Bos et al., 2004b). Despite previous work indicating that gay men may hold lower levels of perceived parenting efficacy because of contextual factors such as homonegative microaggressions and the stigma related to fatherhood broadly (Armesto, 2002; Robinson and Brewster, 2014), the gay fathers in our sample did not report significantly lower levels of perceived parenting competence than any other group. These results are also somewhat aligned with Goldberg and Smith’s (2009) findings regarding parenting competence among LG and heterosexual parents. Although they did find some initial differences with lesbian and heterosexual women reporting greater competence than gay and heterosexual men prior to the adoptive placement of their child, by 3 months post-placement, gay fathers in particular were characterized by the greatest increases in perceived competence as compared to the other parent groups. Taken together, how our results support and differ from prior research (e.g., Armesto, 2002; Goldberg and Smith, 2009) underscore the importance of examining how intersecting identities (e.g., gender, sexual orientation) relate to aspects of family functioning, such as perceived parenting competence. Moreover, our results demonstrate that family functioning can reflect both structure and processes. In these ways, our findings support and extend earlier research about parenting competence among a more diverse sample of both adoptive and sexual minority parents.

Our second hypothesis that earlier mental health symptoms and parenting competence would be associated with later adoption stigma, homonegative microaggressions, and parent–child relationship quality was not supported. Although it is not entirely clear why there was a lack of significant associations among these variables over time, one possibility reflects that the overall levels of mental health symptoms as well as stigma and microaggression experiences were low in this sample. Overall positive adjustment and low levels of stigma and microaggression experiences could also reflect the characteristics of this particular adoptive family sample as being well-resourced in terms of social and practical support, on average (Pinderhughes and Brodzinsky, 2019). Additionally, most of the adoptive parents in this sample lived in urban areas and therefore may have greater access to LG-affirming services (Kinkler and Goldberg, 2011; Goldberg et al., 2013), which may explain why no significant differences emerged in stigma or homonegative microaggressions by coast (East versus West) or urbanicity (rural versus urban). Another possibility is that it is not necessarily the stigma or microaggressions *per se* that relate to individual health and parenting outcomes, but rather the internalization of stigma and the appraisal of microaggression experiences that may have greater impact, as supported by previous research among LG adults, including those who are parents (Goldberg and Smith, 2011; Tornello et al., 2011; Trub et al., 2017). Indeed, the roles of appraisal and internalization of stigma have been posited as among key mechanisms for how minority stress may negatively affect individual adjustment as well as interpersonal relationships (Hatzenbuehler, 2009; Prendergast and MacPhee, 2018), and experiences of stigma represent one external context that could contribute to family stress that spills over into parenting roles and family relationships (Boss et al., 2016). Interestingly, however, greater mental health symptoms were associated with lower perceived parenting competence at both waves, which is consistent with earlier research with adoptive lesbian, gay, and heterosexual parent families (Goldberg and Smith, 2009) and points to underlying connections between individual adjustment and parenting experiences that could have important ramifications for children’s development.

Our third hypothesis related to concurrent associations during middle childhood (W2) among all variables of interest was partially supported by our results. We uncovered positive associations between perceived parenting competence and parent–child relationship quality, assessed at the same time point (W2). This finding is supported by earlier research among heterosexual parent families with biologically related children indicating that parents’ perceived skills have important implications for children’s development (e.g., Martínez-González and Iglesias-García, 2018) and extends it to the first time among an adoptive family sample that includes parents diverse in sexual identity. Parent mental health symptoms and adoption stigma were not significant in statistically predicting parent–child relationship quality assessed at the same time point. This is aligned with our results above and again may reflect the generally low levels of mental health symptoms and adoption stigma among parents in this sample. It could also be that parents are effective in buffering their relationships with their children from their own individual experiences of difficulty or challenge (e.g., Golombok et al., 2018; Green et al., 2019), reflecting family resilience among minority (i.e., adoptive and LG parent) families (Prendergast and MacPhee, 2018); future research could explore these possibilities further.

There was one significant finding related to connections between parents’ homonegative microaggression experiences and children’s perceptions of parent–child relationship quality, both of which were assessed when children were in middle childhood. Among children with LG parents specifically, when parents reported greater current (i.e., within last 6 months) homonegative microaggressions, children described lower quality parent–child relationships. This result emerged even in the context of simultaneous consideration of parent mental health symptoms, parenting competence, and adoption stigma, none of which emerged as significant statistical predictors of parent–child relationship quality at the same time point. This finding is aligned with predictions from family and minority stress theories (McCubbin and Patterson, 1983; Meyer, 2003), indicating connections between sexual minority parents’ experiences of stigma and possible ramifications for the parent–child relationship (Prendergast and MacPhee, 2018). It is possible that LG parents who are experiencing current homonegative microaggressions are also experiencing greater stress and emotional dysregulation as a result, which could interfere with the quality of parents’ relationships with their children; indeed, Hatzenbuehler (2009) describes how interpersonal relationships are one domain in which minority stress may have negative consequences through the effects of resulting psychological distress, cognitive load, and physiological stress. Our finding is also aligned with some related research among children and their LG parents (Bos and Gartrell, 2010; Vyncke et al., 2014; Crouch et al., 2017; Carone et al., 2018; Golombok et al., 2018; Calzo et al., 2019) but extends this work in its theoretical and empirical applications to a sample of adoptive sexual minority parent families and their preadolescent children.

### Limitations, Future Research Directions, and Practice and Policy Implications

Although several strengths of our study include the use of data assessed at two time points as well as multiple informants (i.e., parents and children), it was the case that not all measures were administered at both time points. This limited our ability to assess direction of effects over time. Research incorporating rigorous mixed method longitudinal designs would be advantageous. It is also unclear how well our results would generalize to other samples of adoptive and/or sexual minority parent families. For instance, despite their relevance in previous studies of outcomes among LG and adoptive families, numerous demographic characteristics (i.e., presence of siblings, socioeconomic status, child age) were not found to share statistically significant associations with our variables of interest in this study. This lack of association could reflect the general homogeneity in these demographic variables among this particular sample. Future research is needed to understand more about under what circumstances these variables do serve as important covariates.

Stigma related to sexual orientation and adoption may also operate differently depending on the cultural and sociopolitical context and geographic region in which it occurs (Farr et al., in press), clearly connected with the importance of considering broader external contexts that could contribute to family stress (Boss et al., 2016). For example, LG parent adoptive families living in areas or countries with generally favorable attitudes and policies related to same-gender couples may be provided some protection from the negative effects of stigma—whereas those living in areas characterized by less LG-affirming attitudes or outright discriminatory policies may exacerbate such effects (Kinkler and Goldberg, 2011; Patterson et al., 2013). While our sample is largely representative of other adoptive family samples who pursue private, domestic infant adoption in the US (Pinderhughes and Brodzinsky, 2019), future research would benefit from larger and more diverse samples in terms of geographic location, country of residence, race/ethnicity, socioeconomic status, and pathway to parenthood, among other factors (Fish and Russell, 2018).

Taken together, our findings indicate the value of examining unique contributions of LG-specific processes, such as the role of discrimination and sexual stigma (in this case, parents’ homonegative microaggression experiences) to family outcomes (in this case, quality of parent–child relationships reported by children). Future research would benefit from the incorporation of strong theoretical frameworks, such as combining attention to theories such as family and minority stress, as well as considering a strengths-based approach to processes and outcomes related to resilience among LG parent families (Meyer, 2015; Prendergast and MacPhee, 2018).

These results may be informative to both practices and policies that are supportive of LG parent families in mitigating discrimination and, in turn, supporting individual adjustment of parents and their children. Research has indicated that structural stigma (i.e., governmental, institutional, religious, or other social policies, practices, or laws, as well as cultural and societal norms, community or neighborhood-level attitudes, hate crime rates, etc.) toward sexual minority adults is associated with negative mental health outcomes among sexual minority individuals (Hatzenbuehler, 2014; Herek, 2016), including parents and specifically adoptive parents (Battle and Ashley, 2008; Goldberg and Smith, 2011; Reczek, 2020). With specific regard to clinical, health, and educational practices, our results point to the importance of support from practitioners in cultivating parenting competence among parents in adoptive and sexual minority parent families, especially as parents’ perceptions of their own competence were linked to their children’s perceptions of parent–child relationship quality. Furthermore, our findings underscore the importance of reducing the occurrence and impact of homonegative microaggressions, especially as these were also found to be related to children’s perceptions of closeness with their parents. Practitioners who work with LG parent families could support individual members in learning skills to navigate experiences of stigma in efforts to minimize any harmful effects.

With specific regard to policy implications, there are currently 11 US states with “religious freedom” or “religious exemption” laws that create barriers to fostering and adoption for lesbian, gay, bisexual, transgender, and queer (LGBTQ) prospective or current parents (as well as for LGBTQ children and youth in the foster care system awaiting placement; Movement Advancement Project, 2020). As of November 2019, there is also a proposed rule that would extend these obstacles at the federal level via the US Department of Health and Human Services (Taylor, 2019). Clearly, empirical evidence supports the ability of LG parents to provide loving and effective care to adoptive and foster children (Lavner et al., 2012; Farr, 2017; Patterson, 2017), directly contrasting with existing anti-LGBTQ legislation related to parenting. Our results provide further support regarding the health and well-being of LG adoptive parents and their children, despite facing adversity in the forms of homonegative stigma and discrimination. Rather, our results point to the importance of policies and practices that support LG parent families in managing experiences of discrimination and promoting individual adjustment.

## Conclusion

Consistent with our conceptual framework, our results—derived from both parent and child reports—demonstrate that although adoptive and LG parent families experience stigma, family processes (rather than structure) are most associated with individual outcomes. As recommended by other scholars (e.g., Lo et al., 2019), researchers, policy makers, and practitioners should work together to employ identity-affirming practices to reduce stigma and support adoptive and sexual minority parent family functioning and well-being.

## Data Availability Statement

The datasets generated for this study will not be made publicly available for confidentiality reasons to protect participants. Requests to access the datasets should be directed to the corresponding author.

## Ethics Statement

The studies involving human participants were reviewed and approved by the Institutional Reviews Boards of the University of Virginia, the University of Massachusetts Amherst, and the University of Kentucky. Written informed consent to participate in this study was provided by the participants’ legal guardian/next of kin.

## Author Contributions

RF and CV both made substantial contributions to the conception and design of this work, organized the dataset, contributed to the data analyses and interpretation of results, and read and approved this submitted version. RF collected the data. RF and CV each wrote sections of the manuscript.

## Conflict of Interest

The authors declare that the research was conducted in the absence of any commercial or financial relationships that could be construed as a potential conflict of interest.

## Footnotes

1 Although we are not the first to use the term adoption stigma (e.g., Goldberg et al., 2011; Baden, 2016; Morgan and Langrehr, 2019), we purposefully provide a definition of how we conceptualize it. Baden (2016, p. 1) describes adoption stigma specifically as all the ways (i.e., everyday communication, media messages, books, fairy tales, etc.) that “societal discomfort and judgment about adoption” are conveyed.

2 In W1, five items were inadvertently dropped from the measure administered to participants. As such, we computed a mean score of the remaining 48 items to use in our analyses.

3 This reflects the average alpha of child reports for each parent.
