# Supplementary material for: Stigma Experiences, Mental Health, Perceived Parenting Competence, and Parent–Child Relationships Among Lesbian, Gay, and Heterosexual Adoptive Parents in the United States
Source: Front Psychol. 2020 Mar 30;11:445. doi: 10.3389/fpsyg.2020.00445 (PMC7141157; doi:10.3389/fpsyg.2020.00445)
Supplement: Supplementary file 1 [file Data_Sheet_1.pdf]

Supplementary Material

Summary of Multiple Regression of W1 Variables onto Adoption Stigma

Variable	B	SE(B)	*β*	*t*	*p*
Mental health	.42	.18	.18	2.27	.024*
Competence	.08	.15	.04	.50	.616
R^2			.03		
F(2, 170)		2.62			.076

Note. *p < .05

Summary of Multiple Regression of W2 Variables onto Adoption Stigma

Variable	B	SE(B)	*β*	*t*	*p*
Mental health	.20	.26	.06	.80	.428
Competence	-.20	.14	-.11	-1.42	.158
R^2			.02		
F(2, 169)		1.62			.200

Summary of Multiple Regression of W1 Variables onto Current Homonegative Microaggressions

Variable	B	SE(B)	*β*	*t*	*p*
Mental health	.25	.16	.18	1.52	.132
Competence	.12	.16	.09	.75	.454
R^2			.03		
F(2, 92)		1.16			.318

Summary of Multiple Regression of W1 Variables onto Past Homonegative Microaggressions

Variable	B	SE(B)	*β*	*t*	*p*
Mental health	.22	.24	.11	.91	.368
Competence	.06	.23	.03	.25	.802
R^2			.01		
F(2, 92)		.42			.658

Summary of Multiple Regression of W1 Variables onto Impact Homonegative Microaggressions

Variable	B	SE(B)	*β*	*t*	*p*
Mental health	.31	.29	.12	1.07	.289
Competence	-.03	.28	-.01	-.12	.904
R^2			.02		
$F(2, 92)$		.77			.466

*Summary of Multiple Regression of W1 Variables onto Past*Impact Homonegative Microaggressions*

Variable	B	SE(B)	*β*	*t*	*p*
Mental health	1.32	1.19	.13	1.11	.271
Competence	.12	1.14	.01	.11	.914
R^2			.02		
$F(2, 92)$		.69			.503

*Summary of Multiple Regression of W1 Variables onto Current*Impact Homonegative Microaggressions*

Variable	B	SE(B)	*β*	*t*	*p*
Mental health	1.44	.92	.18	1.56	.122
Competence	.60	.88	.08	.68	.497
R^2			.03		
$F(2, 92)$		1.22			.300

Summary of Multiple Regression of W2 Variables onto Current Homonegative Microaggressions

Variable	B	SE(B)	*β*	*t*	*p*
Mental health	.40	.20	.21	1.97	.052
Competence	-.03	.15	-.02	-.16	.873
R^2			.04		
$F(2, 91)$		2.01			.140

Summary of Multiple Regression of W2 Variables onto Past Homonegative Microaggressions

Variable	B	SE(B)	*β*	*t*	*p*
Mental health	.86	.29	.30	3.01	.003**
Competence	-.21	.22	-.01	-.98	.329
R^2			.11		
$F(2, 91)$		5.40			.006**

Note. $^{**}p < .01$

Summary of Multiple Regression of W2 Variables onto Impact Homonegative Microaggressions

Variable	B	SE(B)	*β*	*t*	*p*
Mental health	.68	.37	.20	1.87	.065
Competence	-.05	.28	-.02	-.18	.861
R^2			.04		
$F(2, 91)$		1.82			.168

*Summary of Multiple Regression of W2 Variables onto Past*Impact Homonegative Microaggressions*

Variable	B	SE(B)	*β*	*t*	*p*
Mental health	3.44	1.49	.24	2.32	.023
Competence	-.76	1.12	-.07	-.68	.498
R^2			.07		
$F(2, 91)$		3.13			.049

*Summary of Multiple Regression of W2 Variables onto Current*Impact Homonegative Microaggressions*

Variable	B	SE(B)	*β*	*t*	*p*
Mental health	2.01	1.17	.18	1.72	.089
Competence	.03	.88	.004	.04	.969
R^2			.03		
$F(2, 91)$		1.49			.231
